# *BRCA1* founder mutations and beyond in the Polish population: A single-institution *BRCA1/2* next-generation sequencing study

**DOI:** 10.1371/journal.pone.0201086

**Published:** 2018-07-24

**Authors:** Artur Kowalik, Monika Siołek, Janusz Kopczyński, Kamila Krawiec, Joanna Kalisz, Sebastian Zięba, Beata Kozak-Klonowska, Elżbieta Wypiórkiewicz, Jowita Furmańczyk, Ewelina Nowak-Ozimek, Małgorzata Chłopek, Paweł Macek, Jolanta Smok-Kalwat, Stanisław Góźdź

**Affiliations:** 1 Department of Molecular Diagnostics, Holycross Cancer Centre, Kielce, Poland; 2 Genetic Clinic, Holycross Cancer Centre, Kielce, Poland; 3 Department of Surgical Pathology, Holycross Cancer Centre, Kielce, Poland; 4 Department of Cancer Epidemiology and Cancer Control, Holycross Cancer Centre, Kielce, Poland; 5 Oncology Clinic, Holycross Cancer Centre, Kielce, Poland; 6 The Faculty of Medicine and Health Sciences, Jan Kochanowski University in Kielce, Poland; CNR, ITALY

## Abstract

Hereditary mutations in *BRCA1/2* genes increase the risk of breast cancer by 60–80% and ovarian cancer by about 20–40% in female carriers. Detection of inherited mutations in asymptomatic carriers allows for the implementation of appropriate preventive measures. *BRCA1/2* genotyping is also important for poly(adenosine diphosphate)-ribose polymerase (PARP) inhibitor administration. This work addresses the need for next-generation sequencing (NGS) technology for the detection of *BRCA1/2* mutations in Poland where until recently mostly founder mutations have been tested, and whether *BRCA* diagnostics should be extended beyond the panel of founder mutations in this population. The study comprises 2931 patients who were referred for genetic counseling and tested for founder and recurrent mutations in *BRCA1* (5382insC (c.5266dupC; p.Gln1756Profs), c.5370C>T (c.5251C>T; p.R1751*), 300T>G (c.181T>G; p.Cys61Gly), 185delAG (c.68_69delAG; p.Glu23Valfs), and 4153delA (c.4035delA; p.Glu1346Lysfs)) by high-resolution melting/Sanger sequencing. A total of 103 (3.5%) mutations were detected, including 53 (51%) in healthy subjects and 50 (49%) in cancer patients. Then, based on more stringent clinical and pedigree criteria, sequencing of all *BRCA1/2* exons was performed in 454 (16%) patients without founder mutations by NGS, which detected 58 mutations (12.8%), 40 (8.8%) of which were pathogenic. In 14 (3.1%) subjects, variants of uncertain significance (VUS) were detected, and in four (0.9%) subjects, the detected mutations were benign. In total, 161 mutations were detected using our two-step algorithm (founder test and NGS), of which 64% were founder mutations, 25% were NGS-detected pathogenic mutations, 9% were VUS, and 2% were benign. In addition, 38 mutations not yet reported in the Polish population were detected. In total, founder mutations accounted for only 64% of all detected mutations, and the remaining mutations (36%) were dispersed across the *BRCA1/2* gene sequences. Thus, in Poland, testing for constitutional mutations in *BRCA1/2* should be carried out in two stages, where NGS is performed in qualifying subjects if founder mutations are not identified.

## Introduction

Hereditary mutations in the *BRCA1* and *BRCA2* genes (*BRCA1/2*) increase the risk of female breast cancer by 60–80% and ovarian cancer by about 20–40% [1–3]. Detection of inherited mutations in asymptomatic carriers allows appropriate preventive actions (imaging, prophylactic surgical procedures) to be taken, which can prevent the development of the disease or increase early detection. In the Polish population, the incidence of founder mutations in breast cancer patients independent of age is around 3% [4]. In Poland, until recently, *BRCA* mutation testing was limited to a few founder mutations [5, 6]. This was mainly due to the relative homogeneity of the Polish population and the very high cost of sequencing of whole *BRCA1/2* genes. Intensive research on *BRCA1/2* mutations in the Polish population was carried out in the 1990s. Thanks to these efforts, the basic founder mutations were identified (*BRCA1*: 5382inC (c.5266dupC), 300T>G (c.181T>G), 185delAG (c.68_69delAG), and 4153delA (c.4035delA)) [7–9]. In comparison to other European populations, a simple and inexpensive test detected 80–90% of *BRCA1/2* mutation carriers in the population [5]. Continuing research detected mutations other than the basic founder mutations, and there were regional patterns in the expression of these mutations [6, 10, 11]. Thanks to the enormous advances in DNA sequencing technology, it has now become possible to study whole genes or gene panels in a quick and economical manner for diagnostic purposes [12]. The study of the whole spectrum of mutations in *BRCA1/2* enables genetic counseling to be optimally implemented and enables the largest group of women at a high risk of developing *BRCA1/2*-related breast or ovarian cancer to be identified. It is also particularly important for the use of poly(adenosine diphosphate)-ribose polymerase (PARP) inhibitors, which are used for the treatment of certain *BRCA1/2*-mutated cancers [13]. However, one of the challenges in interpreting *BRCA1/2* sequencing data is the classification of variants of unknown significance (VUS) and the implementation of genetic counseling in families carrying these variants. There are many practical recommendations in the literature on how VUS should be dealt with clinically [14].

The purpose of the present work is to demonstrate the use of NGS technology for the detection of mutations in *BRCA1/2* in a country with a strong founder effect. It is also an attempt to answer the question of whether *BRCA1/2* diagnostics should extend beyond the panel of founder mutations in the Polish population.

## Materials and methods

This study included 2931 patients who were referred to the Genetic Counseling Outpatient Clinic of the Holycross Cancer Center and were tested for founder and recurrent mutations in *BRCA1* (5382inC (c.5266dupC), 300T>G (c.181T>G), 185delAG (c.68_69delAG), and 4153delA (c.4035delA)). All patients provided written informed consent before DNA testing. All of the study procedures were approved by the Institutional Review Board at Holycross Chamber of Physicians in Kielce (approval number: 20/2018) and performed according to ethical regulation of the Holycross Cancer Centre and the Declaration of Helsinki. All data were fully anonymized prior to access by the authors.

The following criteria were applied to select patients for *BRCA1* testing:

- Women with breast cancer and/or ovarian cancer regardless of age.
- Healthy women who have first- or second-degree female relatives with breast/reproductive organ tumors, as follows: three relatives with breast cancers (BC), one diagnosed before age 50 (HBCss); one relative with BC diagnosed before age 40 or two relatives with BC, one of whom was diagnosed before age 50 (HBCsusp); three relatives with BC and/or ovarian cancer (OC) one diagnosed before age 50 (HBOCss); two relatives with BC and/or OC one diagnosed before age 50 (HBOCsusp); three relatives with OC, one diagnosed before age 50 (HOCss); two relatives with OC or one diagnosed before age 40 (HOCsusp), or a single case of BC/OC reported in the family,
- Women from families with detected mutations in *BRCA1*.

When founder and recurrence mutations were not detected, the following clinical and pedigree criteria were used to select patients for *BRCA1/2* testing by NGS:

- Women with breast cancer and/or ovarian cancer with an additional family history of female tumors (HBCss, HBCsusp, HBOCss, HBOCsusp, HOCss, or HOCsusp; see above).
- Women with breast and/or ovarian cancer with an additional family burden of female tumors (the second breast or ovarian cancer among first-degree relatives, regardless of age).
- Women with breast cancer and ovarian cancer.
- Women with bilateral breast cancer.
- Women with triple-negative breast cancer, regardless of age.
- Woman with ovarian cancer.
- Healthy women with a definitive family history of HBCss, HBOCss, or HOCss.

Using the above criteria, 454 (16%) patients were selected for NGS testing.

### DNA isolation, founder mutation in BRCA1 by genotyping high-resolution melting (HRM)-PCR/Sanger sequencing

DNA was isolated from whole blood. HRM-PCR was performed using Qiagen Type-it PCR Polymerase Mix (Qiagen, Hilden, Germany) in a Rotor Gene Q thermocycler (Qiagen). Reaction results were analyzed using the Rotor-Gene Q Series Software Version 2.2.3 relative to control samples. All mutations detected using the HRM-PCR technique were confirmed using capillary sequencing using the 3130 Capillary Sequencer (Applied Biosystems/ThermoFisher Scientific). Details are described in S1 Supplementary Methods.

### Next-generation sequencing

The libraries were prepared using the Ion AmpliSeq Library Kit, the Ion AmpliSeq *BRCA1/2* Panel, and the Ion Xpress Barcode Adapters Kit, according to the manufacturer's instructions (ThermoFisher Scientific). Sequencing was performed on the Ion Personal Genome Machine (PGM) using the Ion PGM Hi-Q Sequencing Kit and on an Ion S5 sequencer (ThermoFisher Scientific) using the Ion 520 & Ion 530 Kit-Chef. The raw data generated during sequencing was processed using Torrent Server Suite 4.2–5.2 (ThermoFisher Scientific) and the Variant Callerv 4.2–5.2 application. The results were viewed using Integrative Genomics Viewer (IGV; Broad Institute). Additionally, Torrent Server Suite 4.2 generated FASTQ files that were used for analysis by other methods, including the CLC Genomics Workbench, version 7.5.1 (Qiagen) and the Galaxy platform (www.usegalaxy.org). The wANNOVAR program (www.wannovar.usc.edu) was used to annotate the detected variants from Torrent Server Suite and Galaxy. Details are described in S1 Supplementary Methods.

### Classification of the pathogenicity of mutations

Detected mutations were classified based on the information deposited into the Breast Cancer Information Core (BIC) and ClinVar databases. In the case of variants of unknown significance or conflicting results, literature searches and *in silico* analyses using Varsome (https://varsome.com/) were performed. Unfortunately, segregation analysis in the family of the probant was not possible in all cases due to small families, death of family members, and/or lack of consent for genomic analysis S1 Supplementary Methods.

## Results

Among 2931 patients who qualified for screening for founder and recurrent mutations in *BRCA1*, 103 (3.5%) mutations were detected (Table 1). During the study period (3 years), the number of patients who qualified for the test steadily increased. In 2014, 510 patients qualified for testing, and 31 mutations (6.1%) were detected. In 2015, almost twice as many patients (920) qualified for testing as in the previous year, and 26 (2.8%) mutations were detected. In 2016, 1501 patients qualified for testing and 46 mutations (3.1%) were detected (Table 1).

**Table 1:** Frequencies of mutations detected with the screening test targeting founder and recurrent *loci* in *BRCA1* per year and total.

		2014	%	2015	%	2016	%	2014–2016	%
** **	**Number of all tests**	510	100	920	100	1501	100	2931	100
**exon**	**Mutation detected by screening test (BRCA1)**	31	6,1	26	2,8	46	3,1	103	3,5
**20**	**c.5266dupC (p.Gln1756Profs)** **5382insC**	19	3,7	18	2	23	1,5	60	2
**20**	**c.5251C>T (p.Arg1751Ter)** p.R1751*	4	0,8	0	0	11	0,7	15	0,5
**5**	**c.181T>G (p.Cys61Gly)** **[p.C61G]**	6	1,2	7	0,8	11	0,7	24	0,8
**2**	**c.68_69delAG (p.Glu23Valfs)** **185delAG**	2	0,4	0	0	1	0,1	3	0,09
**11**	**c.4035delA (p.Glu1346Lysfs)** **4153delA**	0	0	1	0,1	0	0	1	0,03

* according to Human Genome Variation Society (http://varnomen.hgvs.org/) denotes stop codon (Ter, *)

The 5382insC (c.5266dupC) mutation was detected most often, in 60 (2%) patients, followed by the p.C61G (c.181T>G) mutation in 24 (0.8%) patients and the p.R1751* (c.5251C>T) mutation in 15 (0.5%) patients. The remaining two mutations, 185delAG (c.68_69delAG) and 4153delA (c.4035delA), were detected in only three (0.09%) and one (0.03%) patients, respectively (Table 1). In total, 5382insC (c.5266dupC), p.C61G (c.181T>G), and p.R1751* (c.5251C>T) comprised 96% of the detected mutations (58%, 23%, and 15%, respectively). The remaining 4% consisted of the 185delAG (c.68_69delAG) and 4153delA (c.4035delA) mutations (S1 Fig) (103).

Of the 103 *BRCA1* founder pathogenic mutations, 53 (51%) were detected in healthy subjects and 50 (49%) were detected in cancer patients (Part A in S2 Fig). Pathogenic mutations were detected in 23 (23/50, 46%) patients with breast cancer (age of diagnosis: mean, 51 y; median, 50 y; range, 29–66 y) (Part B in S2 Fig). Among these patients, 16 (70%) patients were diagnosed with triple-negative breast cancer (TNBC), five (22%) with luminal B breast cancer, and one (4%) with human epidermal growth factor receptor (HER)2+ ductal cancer *in situ* (DCIS), and in one (4%) patient, there was no histopathological data on estrogen receptor (ER), progesterone receptor (PR), or HER2 expression (Part D in S2 Fig). Pathogenic mutations were detected in 24 (48%) patients with ovarian cancer (age of diagnosis: mean, 51 y; median, 51 y; range, 35–67) (Part B in S2 Fig). Of these, 18 (75%) were serous tumors, four (17%) were endometriotic, and two (8%) were mucinous (Part C in S2 Fig). Two (4%) patients were diagnosed with both breast cancer and ovarian cancer (one with TNBC and a serous ovarian tumor and one with breast cancer with unknown receptor status and a clear cell ovarian carcinoma). One (2%) man with a *BRCA1* pathogenic mutation was diagnosed with brain cancer (oligoastrocytoma) (Part B in S2 Fig).

Using the criteria described in the Materials and Methods, 454 (16%) individuals in whom we did not detect founder mutations in *BRCA1* were screened for *BRCA1/2* mutations by NGS. Mutations were detected in 58 (12.8%) subjects. Pathogenic mutations were detected in 40 (8.8%) subjects, VUS were detected in 14 (3.1%) subjects, and benign mutations were detected in four (0.9%) subjects (Table 2). Pathogenic mutations accounted for 69% of all 58 mutations detected by NGS (S3 Fig).

**Table 2:** The frequency of mutations detected in *BRCA1/2* with NGS among 454 individuals, per year and total (2014–2016).

	2014	%	2015	%	2016	%	2014–2016	%
**Number of all NGS tests**	81	100	100	100	273	100	454	100
**Detected mutation by NGS (BRCA1&2) including:**	14	17,3	9	9	35	12,8	58	12,8
**Pathogenic**	9	11,1	7	7	24	8,8	40	8,8
**VUS**	2	2,5	2	2	10	3,7	14	3,1
**Benign**	3	3,7	0	0	1	0,4	4	0,9

Among the pathogenic mutations, 21 (21/40, 52.5%) were detected in *BRCA1* and 19 (19/40, 47.5%) were detected in *BRCA2*. The age at the time of the cancer diagnosis was about 5 years lower in carriers of *BRCA1* pathogenic mutations (mean, 46.3 y; median, 46 y; range, 31–77 y) than in *BRCA2* pathogenic mutation carriers (mean 49.6 y; median, 51 y; range, 32–65 y).

Regarding the *BRCA1* pathogenic mutations, 10 were frameshift mutations, eight were nonsense mutations, and three were missense mutations. In the case of *BRCA2* pathogenic mutations, 12 were frameshift mutations, six were nonsense mutations, and one mutation disrupted normal splicing (S2 Table). Mutations were detected in four (4/40, 10%) healthy subjects and in 36 (36/40, 90%) cancer patients (Part A in S4 Fig). Mutations were detected in 19 (19/36, 53%) patients with breast cancer (age at diagnosis: mean, 45 y; median, 44.5 y; range, 31–77 y) (Part B in S4 Fig). Among breast cancers, mutations were detected in patients with the following diagnoses: seven (7/19; 37%) TNBC, four (4/19; 21%) luminal B, three (3/19; 16%) luminal A or B (no data on the Ki67 proliferation index), two (2/19; 10.5%) luminal A, two (2/19; 10.5%) DCIS, and one (1/19; 5%) invasive ductal carcinoma (IDC) with no data on receptor status (Part D in S4 Fig). Mutations were also detected in 16 (16/36, 44%) patients with ovarian cancer (age at diagnosis: mean, 51 y; median, 51 y; range, 42–65 y) (Part D in S4 Fig). The majority of the patients had serous tumors (14/16, 87%), and the remainder had endometrioid cancers (2/16, 13%) (Part C in S4 Fig). One patient (1/36, 3%) was diagnosed with breast cancer (luminal A subtype) at 47 years of age and was diagnosed 7 years later with serous ovarian cancer (Part B in S4 Fig).

In all 14 cases in which VUS were detected, the VUS were not accompanied by other pathogenic mutations in *BRCA1* or *BRCA2* (S3 Table). All VUS were caused by missense mutations in one nucleotide. In total, 11 different VUS were detected (S3 Table). The *BRCA2* p.N3124I mutation was detected in three patients treated for breast cancer (two with luminal A or B and one with TNBC), and the *BRCA2* p.R2336H mutation was detected in two unrelated probands (one TNBC patient and one healthy subject). Patients with the p.N3124I mutation came from families with multiple cases breast cancer (HBCs and HBCss) and families with breast and ovarian cancer (HBOCs). Similarly, the carriers of the p.R2336H mutation were from families with multiple breast cancer cases (HBCss) (S3 Table).

VUS were detected in 12 (12/14, 86%) patients with cancer and two (2/14; 14%) healthy subjects (Part A in S5 Fig). Among the carriers of the VUS mutation, eight (8/12, 67%) patients had breast cancer (of which two cases were bilateral), and four (4/12, 33%) patients had ovarian cancer (Part B in S5 Fig). Of the ovarian cancer cases, three (3/4; 75%) were serous carcinomas and one (1/4; 25%) was a clear cell carcinoma (Part C in S5 Fig). VUS was detected in two (2/8; 25%) patients with TNBC, two patients (2/8; 25%) with luminal B breast cancer, one patient (1/8; 12.5%) with luminal A and one patient (1/8; 12.5%) with a HER2+ tumor. In two (2/8, 25%) cases, the luminal A and B subtypes could not be distinguished due to the lack of data on the Ki67 proliferation index (Part D in S5 Fig and S5 Table).

To assess the pathogenicity of VUS, we conducted an *in silico* analysis using the algorithms available on the Varsome website (https://varsome.com/) (S4 Table). For comparison, S4 Table shows the results of the analysis of the known founder mutation in *BRCA1* considered to be pathogenic (p.C61G) and the detected VUS. At least four VUS in *BRCA2* (p.G3076E, p.Y3092C, p.R2336H, and p.N3124I) showed very similar results to the p.C61G founder mutation in *BRCA1*. Interestingly, two of these mutations (p.N3124I and p.R2336H) were detected in subjects (see above) from families with multiple cases of breast or ovarian cancer and breast cancer (S3 Table). However, in these families, the segregation of the variant and the association with breast/ovarian cancer could not be demonstrated. In addition, the variant could not be tracked back to the parental lines since other affected family members did not consent for testing or were deceased.

In four subjects, benign mutations were detected (p.K3326X, p.I3412V, and p.T2515I) (S5 Table). TNBC was diagnosed in both carriers of the p.K3326X mutation. By contrast, the patient with the p.I3412V mutation was diagnosed with infiltrating breast cancer of the luminal B subtype (HER2-). The p.T2515I mutation was detected in a healthy subject who comes from a family with many breast cancer cases (HBCss).

**Fig 1:**
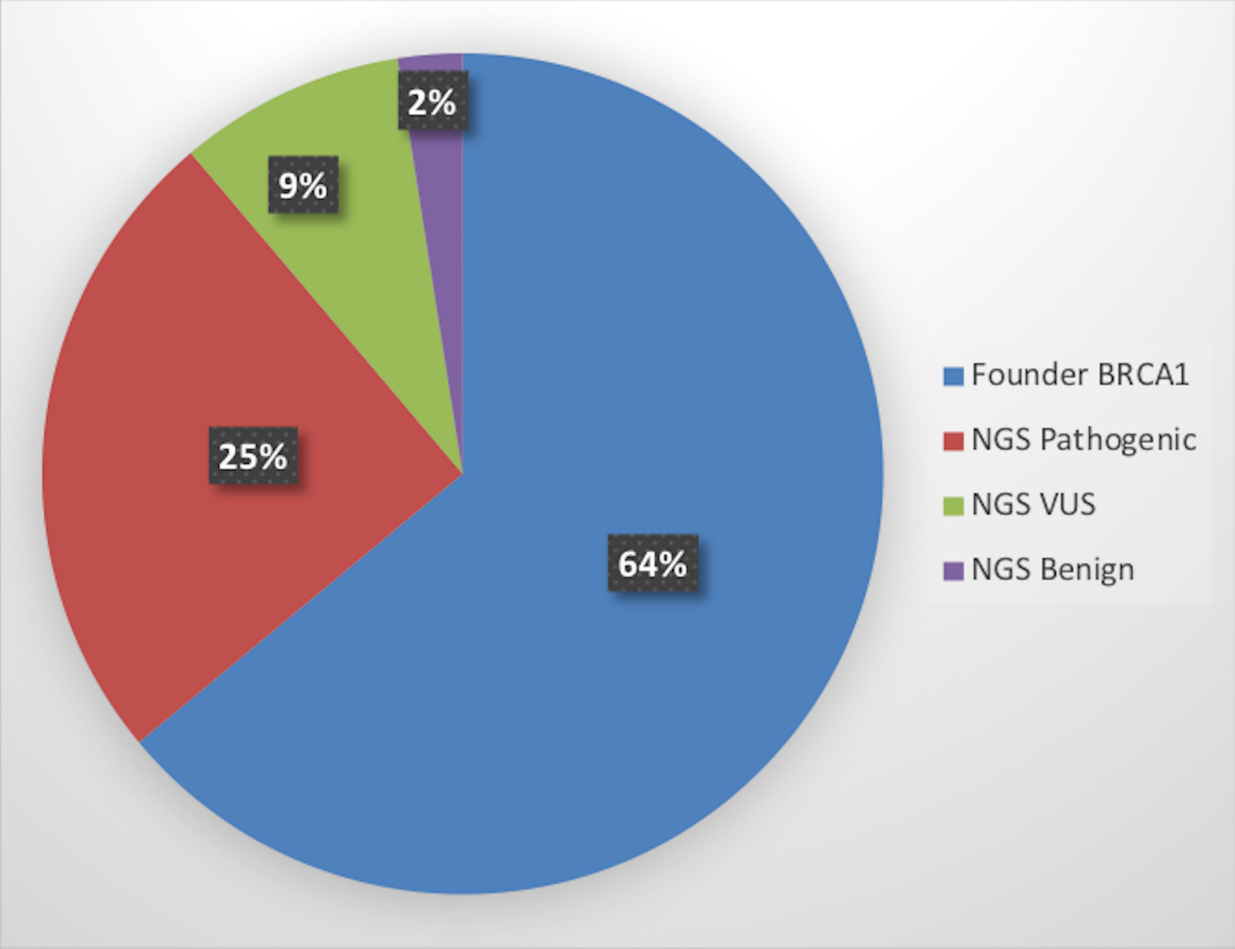
Percentage share of all detected mutations with both the screening test (103) and NGS (58): Pathogenic (40), VUS (14), benign (4).

In total, 161 mutations were detected using our two-step algorithm (initial screening for founder mutations, followed by NGS). Of the detected mutations, 64% were founder and recurrent pathogenic mutations. Of the mutations detected by NGS, pathogenic mutations accounted for 25%, while 9% were VUS and 2% were benign (Fig 1). Is should be mentioned that maintaining two-step approach (founder mutation detection + targeted BRCA1/2 NGS) versus using only a targeted BRCA1/2 NGS-based technology is up to 50% cheaper.

**Fig 2:**
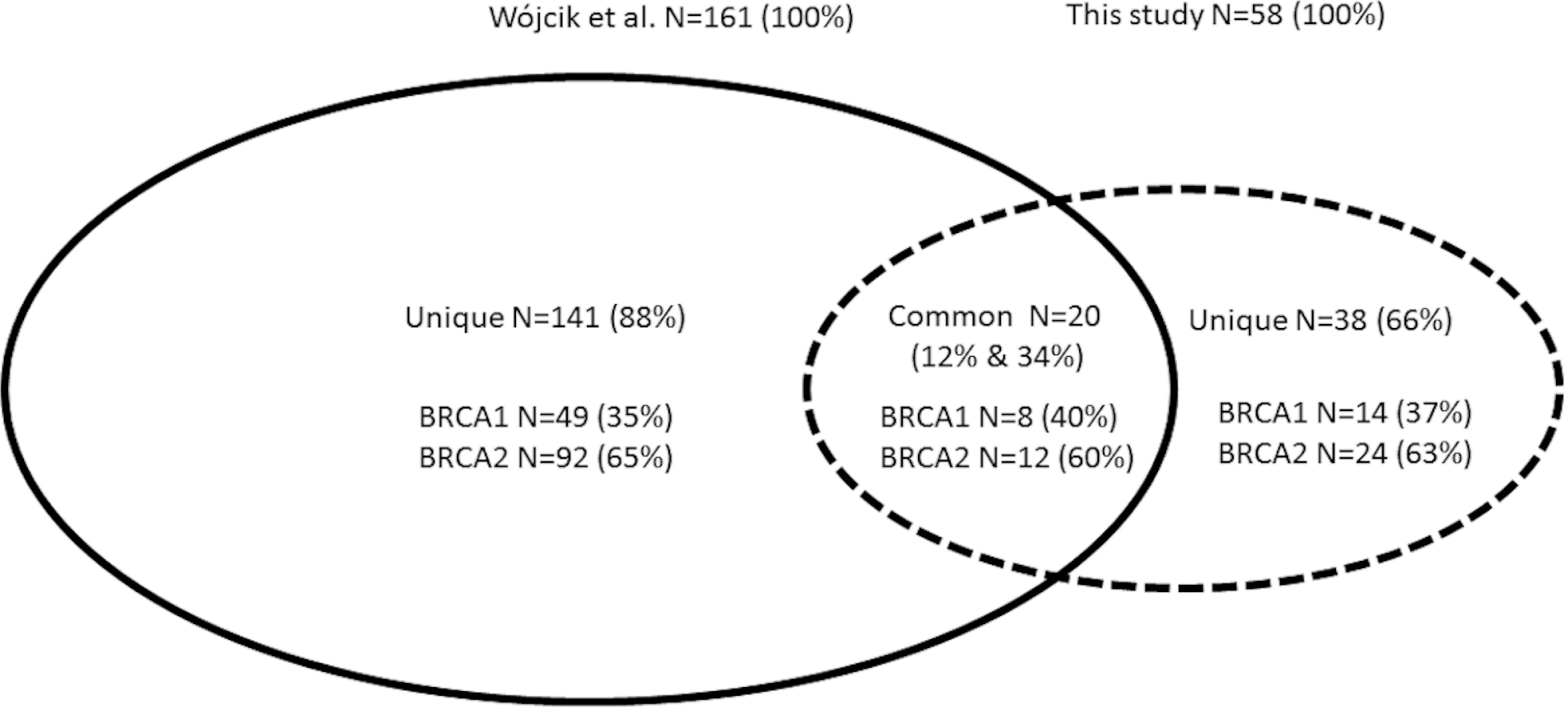
Comparison of the frequency of detected non-founder mutations between the meta-analysis of previously published studies in the Polish population (meta-analysis [15]) and non-founder mutations detected in the current work.

Our results obtained using NGS were compared with data published in a meta-analysis of all studies on non-founder mutations performed on the Polish population in the years 2000–2015 [15]. In this meta-analysis [15], the researchers described a total of 161 mutations in *BRCA1/2*, 20 (20/161, 12%) of which were also detected in the present study (“common” mutations) (Fig 2). In our study, common mutations accounted for 34% (20/58) of all mutations detected (Fig 2). In total, out of the 199 mutations described in our study or in the meta-analysis, 141 (71%) were unique mutations described in the meta-analysis [15], 38 (19%) were unique mutations detected only in the present work, and 20 (10%) were common mutations detected in both studies (S6 Fig). For both *BRCA* genes, these ratios are identical. To summarize, in the current study, 38 mutations were detected that have not yet been reported in the Polish population (S6 Fig).

## Discussion

The observed steady increase in patients who qualified for genetic testing during the study period (2014–2016) is associated with increasing awareness among doctors and patients of regarding the role of genetic testing in oncology (also with the "Angelina Jolie effect" [15].) This increase in genetic testing also correlates with the worldwide introduction of PARP inhibitors for the treatment of cancers with mutations in *BRCA1/2* [13].

It is noteworthy that in the population we studied, the 4153delA (c.4035delA, p.Glu1346Lysfs) pathogenic mutation, which is included in the founder panel for the Polish population, was detected in only one patient [5]. By contrast, over 90% of detected mutations consisted of the two founder mutations 5382insC (c.5266dupC; p.Gln1756Profs) and 300T>G (c.181T>G; p.C61G) and the regionally-occurring recurrent mutation c.5370C>T (c.5251C>T; p.R1751X (S1 Fig). This highlights regional differences in the prevalence of founder mutations in the Polish population.

Comparing the demographics of the ovarian cancer patients included in this study, the average age of diagnosis in carriers of founder pathogenic mutations and pathogenic mutations detected by NGS was 51 years, which is slightly higher than that reported by Wójcik et al. (46.7y) [16]. However, the average age of the carriers was similar to that reported in the American population (50y for *BRCA1* and 56.5y for *BRCA2*) [17]. The most common histological subtype of ovarian cancer in patients in whom pathogenic mutations were detected was the serous subtype (75–87%), followed by endometroid subtype (13–17%) and the mucinous subtype (8%) (S2 and S4 Figs). This is consistent with the rates reported in the Polish population as well as in other populations (44–67%, 12–37%, and 1–4% respectively) [18–21].

The average age diagnosis of breast cancer in the carriers of founder pathogenic mutations was 51 years. A similar average age (48.5 y) is reported by Wójcik et al. [16]. However, this is 10 years older than in the American population, in which the average age of breast cancer diagnosis in *BRCA1/2* pathogenic mutation carriers is 41 years [17]. These differences may result from the sizes of the study groups, population differences, or the algorithm used to select patients for genetic testing, or this may reflect the greater effectiveness of screening for breast cancer in the US than in Poland. Interestingly, in carriers of non-founder pathogenic mutations (NGS-detected), the average age of breast cancer diagnosis was 45 years. This is probably the effect of the algorithm used to select patients for testing NGS mutations (see Material and Methods).

Histologically, the tumors in carriers of founder pathogenic mutations (*BRCA1*) were most commonly of the TNBC and luminal B subtypes (S2 Fig). The relative frequencies of the breast cancer subtypes among carriers of pathogenic non-founder mutations were similar to the frequencies among carriers of pathogenic founder mutations (most frequently TNBC and luminal B, followed by luminal A) (S4 Fig). Among carriers of mutations in *BRCA1/2*, there is a higher probability of occurrence of breast cancer of the TNBC phenotype. The incidence of TNBC in patients with mutations in *BRCA1/2* is approximately 20% [22].

In the overwhelming majority of cases, the frameshift (F) or nonsense (N) mutations were unequivocally classified as pathogenic and do not cause difficulties in interpretation and genetic counseling. Missense (M) mutation cause more difficulties in interpretation. In the group of mutations detected by NGS classified as pathogenic were two missense mutations in *BRCA1* detected in three subjects: 2x p.C39R and 1x p.R1699W. In our work, p.C39R mutations were detected in a woman with TNBC and the healthy carrier. Mutation p.R1699W was detected in a breast cancer of luminal A or B subtype (S2 Table).

Previous studies have indicated that all mutations affecting the cysteines in the RING domain (including codon p.C39) of BRCA1 have an effect on the proper functioning of this protein by reducing its homologous recombination (HR) activity. The introduction of the p.C39Y mutation into a cell line decreased HR by over 80%. The p.C39Y mutation also prevented the formation of heterodimers with BRCA1-associated RING domain 1 (BARD1), which stabilizes the BRCA1 protein [23].

A previous analysis using a multifactorial probability-based model classified the p.C39Y mutation as pathogenic [24]. The mutation p.R1699W has been associated with malignant phyllodes tumors of the breast [25]. Recently, another mutation in the same codon of *BRCA1* (p.R1699Q) was described as an intermediate risk factor in the development of breast and ovarian cancer. For carriers of this mutation, annual mammography from 40 years of age is recommended. Bilateral salpingo-oophorectomy (BSO) should also be considered based on pedigree analysis [26]. The p.R1699W variant is also classified as pathogenic in the Evidence-Based Network for the Interpretation of Germline Mutant Alleles (ENIGMA) consortium database (http://brcaexchange.org/) as well as using the multifactorial probability-based model [24].

Assessing the pathogenicity of VUS in *BRCA1* and *BRCA2* poses a serious challenge for the interpretation and implementation of genetic counseling and radical preventive strategies. To unify interpretations of these mutations, international consortia such as ENIGMA (https://enigmaconsortium.org/) were established, and recommendations have been issued [14, 27]. In our work, we detected 11 mutations that are currently considered VUS in 14 subjects (S3 Table). The *in silico* and pedigree analysis indicated that at least two of these variants (p.N3124I and p.R2336H in *BRCA2*) can be classified as probably pathogenic (S4 Table). In addition, the mutation p.N3124I has been described previously in the Polish population in a man with breast cancer [28] and in six Polish patients from families with breast and ovarian cancers [29]. Recently, another research group reported this mutation as pathogenic, presenting pedigree analysis from seven families as well as a population analysis (the absence of the p.N3124I mutation in 3126 healthy subjects) [30]. According to American College of Medical Genetics (ACMG) guidelines [25], the mutation p.N3124I should be reclassified as probably pathogenic [fulfilling ACMG criteria PS4 (higher prevalence in affected individuals versus controls), PP3 (computational evidence), and PP4 (highly specific individual phenotype or family history)] and associated with breast and ovarian cancer.

By contrast, the mutation p.R2336H can lead to splicing disruption [exon 13 deletion, with a shortened protein length (p.Gly2313AlafsX31)] [31]. BRCA2 with the p.R2336H mutation exhibits disrupted HR and therefore reduced DNA repair. This mutation lies in the binding domain of the Fanconi anemia complementation group D2 (FANCD2) and CG (FANCG) proteins; thus interactions with these proteins may be impaired. In addition, cells with the p.R2336H mutation show reduced expression of full-length BRCA2 protein [32]. Taken together, the p.R2336H mutation significantly reduces the functionality of the BRCA2 protein and thus its ability to repair damage in DNA correctly and efficiently. It can therefore cause a moderate risk of cancer. A meta-analysis of population studies looking at the frequency of this variant may more accurately determine this risk. By contrast, the mutations p.3076E and p.Y3092C in *BRCA2* were detected in patients with luminal B and luminal A breast cancer, respectively, and had not been previously reported in the Polish population. *In silico* analysis predicted both of these mutations to be as pathogenic (S4 Table). However, recently, the p.Y3092C mutation was classified as probably benign using the multifactorial probability-based model [24]. In the future, one should expect the use of global mutational signatures for the evaluation of the pathogenicity of VUS [33, 34].

The challenges of classification apply not only to VUS, but also to nonsense variants in which the last amino acids in the BRCA proteins are lost. An example of such a variant is BRCA2 p.K3326X, which is classified as benign in the ClinVar and Breast Cancer Information Core (BIC) databases. Additionally, in a previous report, 18 breast cancer tissue samples harbored this variant but did not show elevated levels of global *mutation signature 3*, characteristic of tumors with biallelic inactivation of *BRCA1/2*. However, the authors didn’t clearly describe *BRCA1/2* allelic status in these cases [34]. A recently published multicenter study indicates that carriers of this variant have an increased risk of developing breast cancer and ovarian cancer independently of other pathogenic variants in *BRCA2* [35]. In our study group, both carriers of this mutation had been diagnosed with TNBC (S5 Table).

Based on these results, we estimate that in the Polish population, founder mutations constitute 64–70% of all *BRCA1/2* mutations (Fig 1). This figure is approximately 20% less than early estimates (86%) [5]. In addition, Ratajska et al. recently detected a similar percentage of founder mutations (65%) in Polish women with ovarian cancer using the NGS method [20]. An even lower percentage of founder mutations (48%) was observed by Wójcik et al. [16]. In connection with the above data, it is necessary to introduce NGS-based testing of selected subjects in the Polish population in order to optimally identify carriers of pathogenic variants of *BRCA1/2*.

In our study, 19% of the detected mutations were hitherto unreported in the Polish population (S6 Fig), but have been described in other populations around the world (ClinVar, BIC). With the increased access to *BRCA1/2* genetic testing by NGS, we should expect to describe new mutations in the Polish population. One of the caveats of this work is the lack of assessment of large *BRCA1/2* gene rearrangements. The frequency of such aberrations in Polish populations with familial breast and ovarian cancers is not high (around 5%) [11]. In the future, this could be accomplished with NGS, which, based on the coverage data, should allow for detection of large rearrangements (entire exons, parts of genes, or entire genes) alongside the detection of mutations [36]. Another issue is the lack of evaluation of the occurrence of mutations in other genes coding for proteins functionally linked to BRCA1/2 in the process of repairing DNA damage [37].

In the case of detected pathogenic founder mutations, 23% (24/103) of carriers were diagnosed with ovarian cancer. In addition, 40% (16/40) of carriers of pathogenic mutations detected with NGS suffered from ovarian cancer. Considering all (40 = 16+24) patients with ovarian cancer, applying a two-step algorithm enabled detection of 40% (16/40) more patients with *BRCA1/2* mutations potentially benefiting from PARP inhibitor therapy.

Interestingly, using selection criteria for *BRCA1/2* mutation testing by the NGS, we would identify only 69% (61/103) probands for testing from among the founder mutation carriers. On the other hand, we would not qualify 31% (32/103) probands, the majority of whom are healthy people without strong family history of cancer. Above calculations strongly support our two step diagnostic algorithm *BRCA1/2* mutation testing.

In conclusion, in this work, founder mutations constituted 64–70% of all detected mutations. The remaining mutations (approximately 30–36%) were dispersed along the *BRCA1/2* sequences. These results indicate that, in Poland, the detection of mutations in *BRCA1/2* should be carried out in two stages, where NGS is performed if founder mutations are not identified.

## Supporting information

S1 Supplementary Methods (DOCX)

S1 Fig Percentage share (%) of all 103 mutations detected by the screening test. (TIF)

S2 Fig Clinical characterization of the 103 founder and recurrent BRCA1 mutation carriers identified by the screening test. (TIF)

S3 Fig Percentage share (%) of all 58 mutations detected by NGS in the *BRCA1/2*. (TIF)

S4 Fig Clinical characterization of the 40 *BRCA1/2* mutation carriers detected with NGS. (TIF)

S5 Fig Clinical characterization of the 14 BRCA1/2 VUS carriers identified with NGS. (TIF)

S6 Fig Percentage share all BRCA1/ 2 mutations in the Polish populations detected with NGS. (TIF)

S1 Table The primer sequences used for *BRCA1* founder mutations analysis by HRM-PCR/Sanger Sequencing. (XLSX)

S2 Table Clinical and pathological characteristics of the pathogenic mutation carriers detected with NGS. (XLSX)

S3 Table Clinical and pathological characteristics of the VUS carriers identified with NGS. (XLSX)

S4 Table *In silico* analysis of VUS using Varsome (https://varsome.com/). (XLSX)

S5 Table Clinical and pathological characteristics of benign mutation carriers identified with NGS. (XLSX)
