# Supplementary material for: BRCA1 founder mutations and beyond in the Polish population: A single-institution BRCA1/2 next-generation sequencing study
Source: PLoS One. 2018 Jul 24;13(7):e0201086. doi: 10.1371/journal.pone.0201086 (PMC6057642; doi:10.1371/journal.pone.0201086)
Supplement: S1 Supplementary Methods — (DOCX) [file pone.0201086.s001.docx]

**Supplementary Methods**

**DNA isolation**

Whole blood collected in EDTA tubes was used for DNA isolation. DNA was isolated from 100 μl of whole blood within 12 hours of collection using the Micro AX Blood Gravity Kit (A&A Biotechnology, Gdańsk, Poland). The DNA was eluted in a volume of 120 μl of buffer E.

**High-Resolution Melting (HRM)-PCR/Sanger Sequencing**

**Detection of mutations in the *BRCA1* gene by HRM-PCR**

We have been using HRM for 10 years. Before we introduced HRM to detect the founder mutations in BRCA1, this method was validated based on control samples. In addition, several hundred samples were genotyped using both HRM and Sanger technologies. We obtained 100% concordance in mutation detection using both technologies. To confirm the presence of a mutation detected by HRM with Sanger, we usually select more samples for confirmatory Sanger Sequencing than it could be estimated by analyzing HRM curves, which protects us against false negative results. Based on above facts we are sure that screened founder and recurrent mutations in BRCA1 (5382insC- c.5266dupC (p.Gln1756Profs), c.5370C>T- c.5251C>T (p.R1751*), 300T>G - c.181T>G (p.Cys61Gly), 185delAG- c.68_69delAG (p.Glu23Valfs), and 4153delA- c.4035delA (p.Glu1346Lysfs)) are all detected with very high confidence.

PCR was performed using a mixture of 7 μl Qiagen Type-it PCR Polymerase Mix (Qiagen, Hilden, Germany), 5 μl water, 1 μl of each primer (S1 Table), and 1 μl template DNA. The reaction was carried out in a Rotor Gene Q thermocycler (Qiagen). The reaction conditions were: 95 °C for 5 min; 40 cycles of 95 °C for 10 s, 67 °C for 30 s (touchdown of 1 °C/cycle for 10 cycles), and 72 °C for 20 s; and one cycle of 95 °C for 10 s and 40 °C for 20 s. After completion of the PCR reaction, the HRM melting reaction was performed on a Rotor Gene Q thermocycler (Qiagen). The HRM range for the examined exons in the *BRCA1* gene was 75-87 °C. Reaction results were analyzed using the Rotor-Gene Q Series Software Version 2.2.3 relative to control samples. The melting curves profiles of the tested samples were compared with the melting curves of the non-mutated and mutated control samples.

All mutations detected using the HRM-PCR technique were confirmed using capillary sequencing. Curves diverging in shape from the control curve (WT) were verified by capillary sequencing. The Sanger sequencing reaction used PCR amplification products that were purified using 10 U of exonuclease I (EN 0582) and 1 U of phosphatase Fast-AP (EF 0651) (both from ThermoFisher Scientific, Waltham, MA). The reaction was incubated for 15 min at 37 °C, followed by 20 min at 80 °C. Sequencing reactions were performed using forward and reverse sequence-specific primers (S1 Table) and the ABI PRISM Big Dye Terminator Kit, version 3.1 (catalogue number 4337450, Applied Biosystems/ThermoFisher Scientific), according to the manufacturer’s instructions. The sequencing results were analyzed using the 3130 Capillary Sequencer (Applied Biosystems/ThermoFisher Scientific). The generated sequences were compared to the reference sequence using the NCBI Blast Nucleotide program.

**Next-generation sequencing**

**Library preparation**

The DNA concentration was measured using a Qubit I fluorometer and the dsDNA High-Sensitivity Assay Kit (ThermoFisher Scientific). The DNA was diluted to a concentration of 10 ng/μl. The libraries were prepared using the Ion AmpliSeq Library Kit, the Ion AmpliSeq *BRCA1/2* Panel, and the Ion Xpress Barcode Adapters Kit, according to the manufacturer's instructions (ThermoFisher Scientific). The *BRCA1/2* panel used for the multiplex PCR contains 167 primer pairs, amplifying an area of ​​16.25 kb that contains all the coding sequences of *BRCA1/2*. The primers were divided into three separate sets containing 55–56 primers each. For each of the samples tested, three separate multiplex PCR reactions were performed. The resulting multiplex PCR products were combined and then subjected to partial enzymatic digestion to remove primer sequences. Next, using the IonXpress Barcode Adapters Kit (ThermoFisher Scientific), adapters were enzymatically attached to both ends of the PCR products. One of the adapters contained the barcodes, which enabled identification of sequences from a given patient from among a mixture of libraries. Then, prepared libraries were purified using Agencourt AMPure XP (Beckman Coulter Genomics) according to the manufacturer's instructions.

**Preparation of clonally amplified templates for sequencing with the Personal Genome Machine**

The concentrations of the prepared libraries were measured by real-time PCR using the Ion Library Quantitation Kit (ThermoFisher Scientific). Based on the values ​​obtained, all prepared libraries were diluted to 20 pM. The 16 libraries were then mixed and clonally amplified templates were prepared by emulsion PCR (emPCR) using the Ion OneTouch 2 System and the Ion PGM Hi-Q OT2 Kit according to the manufacturer's instructions. The quality of the clonally amplified libraries was measured using the Ion Sphere Quality Control Kit (ThermoFisher Scientific) on a Qubit fluorometer. Next, the clonally amplified library was enriched and purified using MyOne Streptavidin C1 Dynabeads and the Ion OneTouch ES system, according to the manufacturer's instructions. During this process, template negative spheres, low-quality, and incomplete templates were removed.

**Preparation of clonally amplified templates for sequencing using the IonChef**

The concentrations of the prepared libraries were measured by real-time PCR using the Ion Library Quantitation Kit (ThermoFisher Scientific), and all prepared libraries were diluted to a concentration of 30 pM. Then, barcoded libraries were mixed. emPCR and enrichment were performed using IonChef (ThermoFisher Scientific) and the Ion 520 & Ion 530 Kit-Chef (ThermoFisher Scientific).

**Sequencing with the Personal Genome Machine (PGM)**

Sequencing was performed on the Ion Personal Genome Machine (PGM) using the Ion PGM Hi-Q Sequencing Kit. The 316 chip was used. Sequencing was done according to the manufacturer's instructions.

**Sequencing with the Ion Chef System**

Sequencing was carried out on an Ion S5 sequencer (ThermoFisher Scientific) using the Ion 520 & Ion 530 Kit-Chef. Two 530 chip was used. Sequencing was done according to the manufacturer's instructions.

**Data analysis**

The raw data generated during sequencing was processed using Torrent Server Suite 4.2-5.2 (ThermoFisher Scientific). The obtained sequences were mapped to the reference sequence of the human genome (hg19). Searching for single nucleotide polymoprhisms (SNPs) was carried out using the Variant Callerv 4.2-5.2 application, which is part of Torrent Server Suite 4.2-5.2. The manufacturer-recommended parameters for AmpliSeq Germline were used: minimum allele frequency, 0.1; minimum quality, 10; and minimum coverage, 10. The results were viewed using Integrative Genomics Viewer (IGV; Broad Institute). Additionally, Torrent Server Suite 4.2 generated FASTQ files that were used for analysis by other methods, including the CLC Genomics Workbench, version 7.5.1 (Qiagen) and the Galaxy platform (www.usegalaxy.org). The basic parameters used in the analysis by CLC were as follows: minimum allele frequency, 0.1; minimum quality, 10; and minimum coverage, 40. The following parameters were used for Galaxy: minimum allele frequency, 0.10; minimum quality, 25; and minimum coverage, 40. The wANNOVAR program (www.wannovar.usc.edu) was used to annotate the detected variants from Torrent Server Suite and Galaxy.
