# Supplementary figures and images for: BRCA1 founder mutations and beyond in the Polish population: A single-institution BRCA1/2 next-generation sequencing study
Source: PLoS One. 2018 Jul 24;13(7):e0201086. doi: 10.1371/journal.pone.0201086 (PMC6057642; doi:10.1371/journal.pone.0201086)

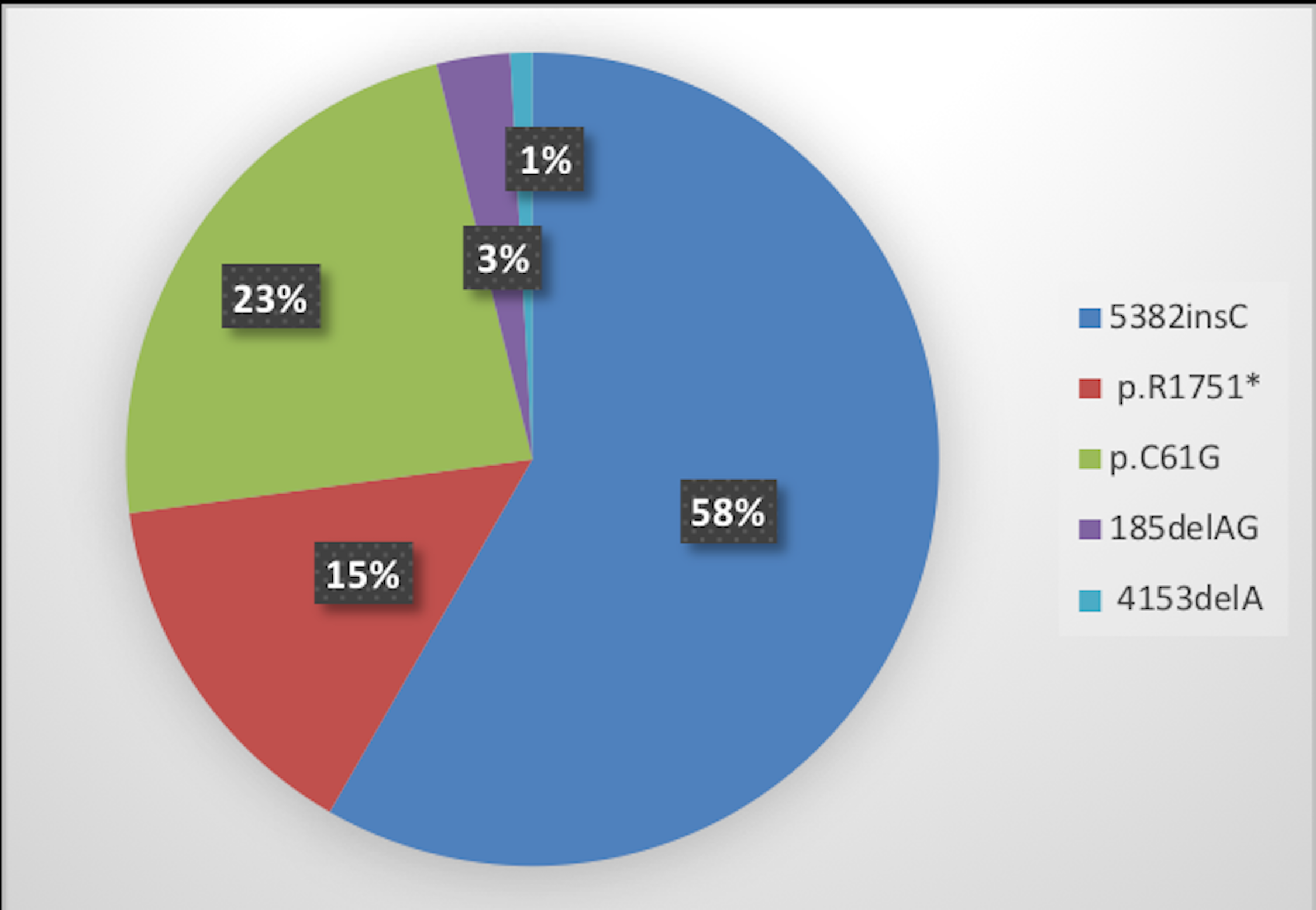

Supplement: S1 Fig — (TIF) [file pone.0201086.s002.tif]

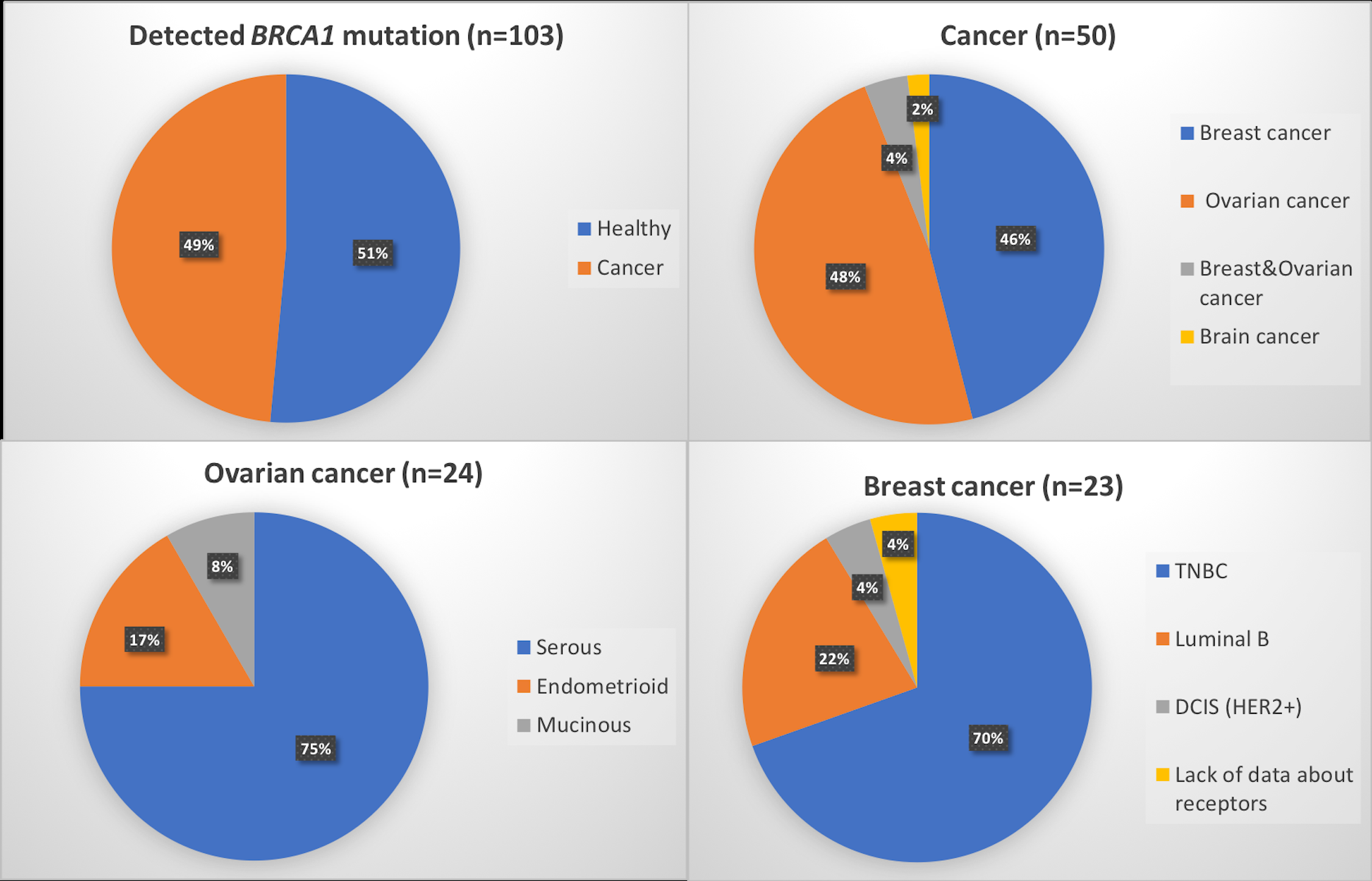

Supplement: S2 Fig — (TIF) [file pone.0201086.s003.tif]

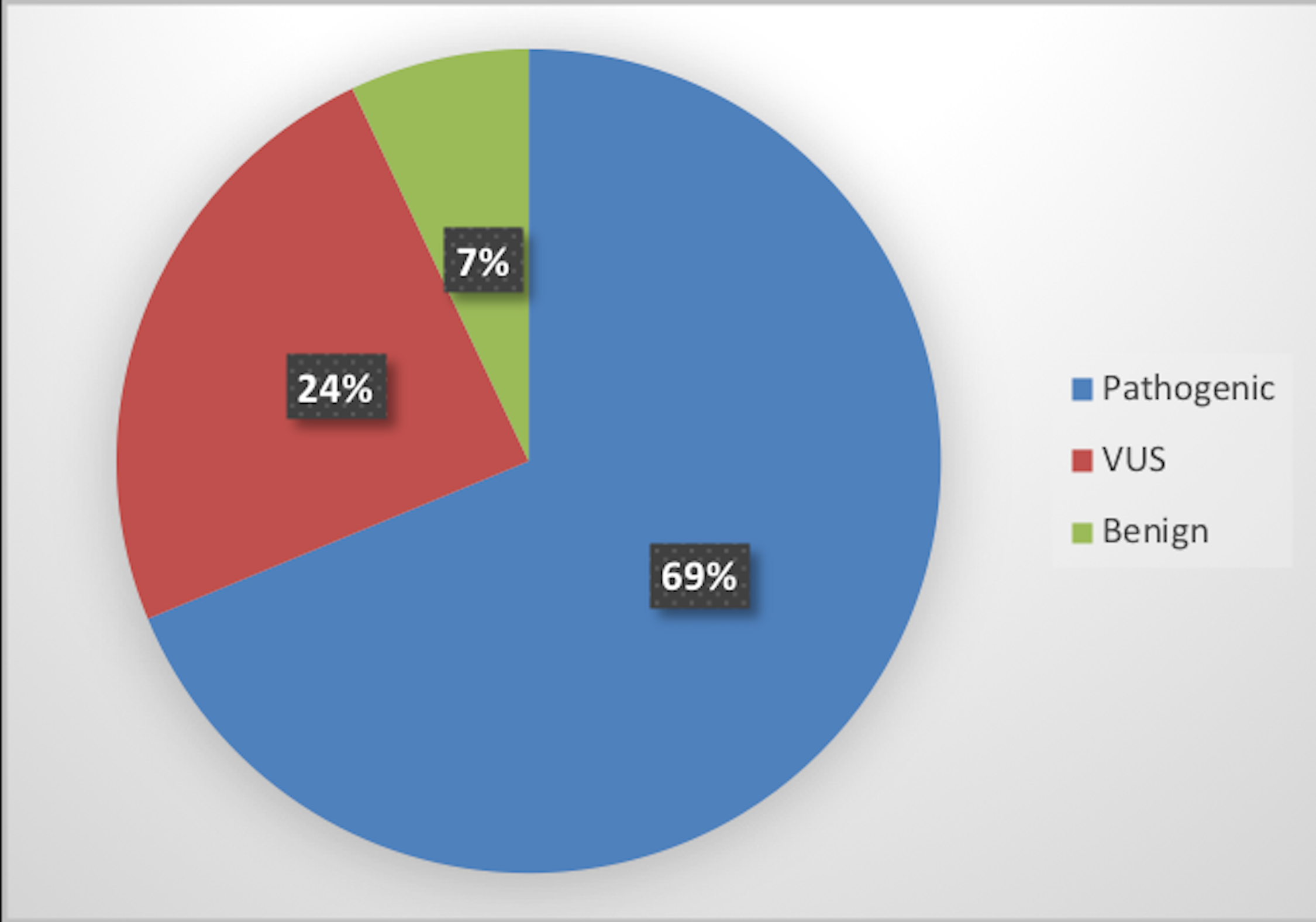

Supplement: S3 Fig — (TIF) [file pone.0201086.s004.tif]

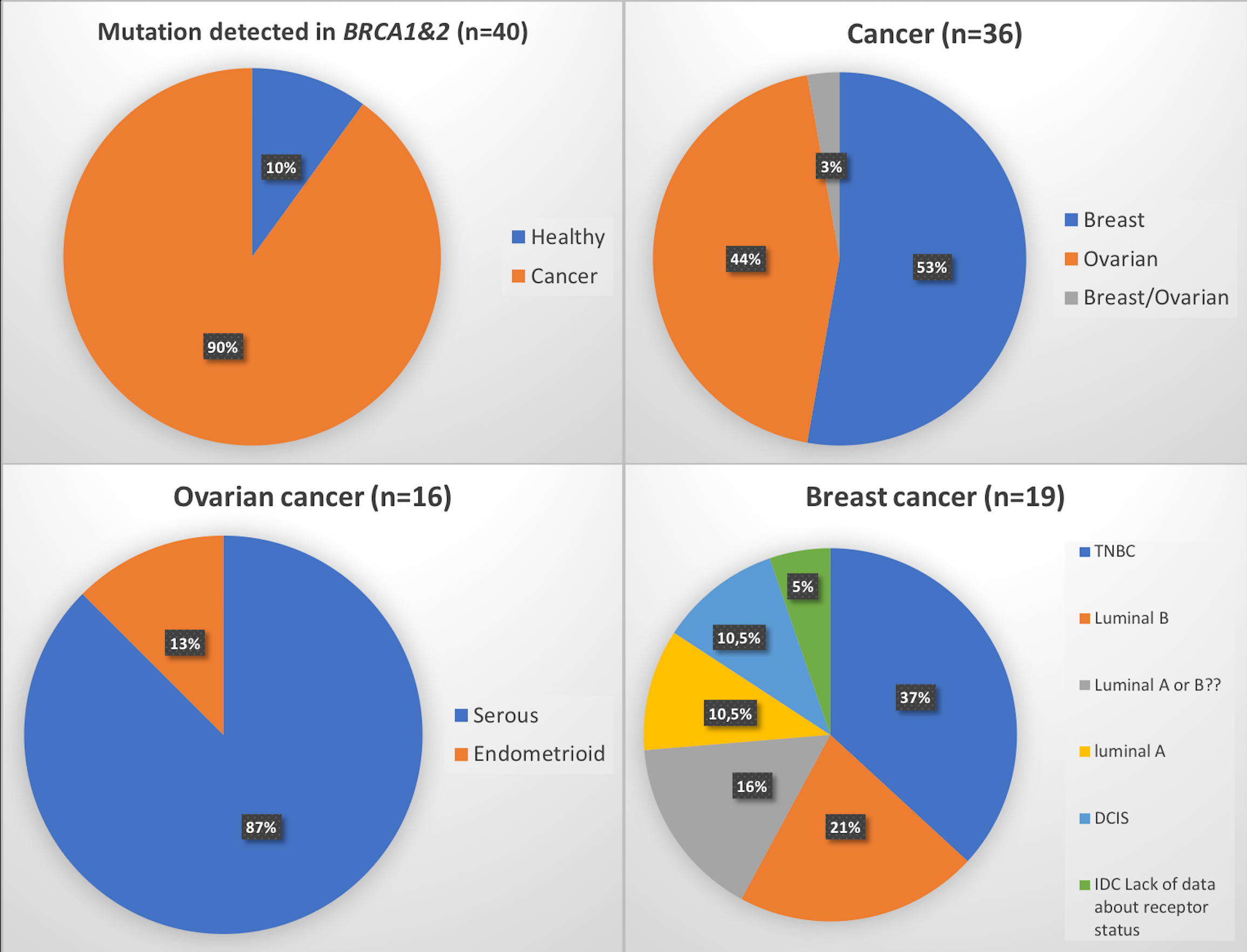

Supplement: S4 Fig — (TIF) [file pone.0201086.s005.tif]

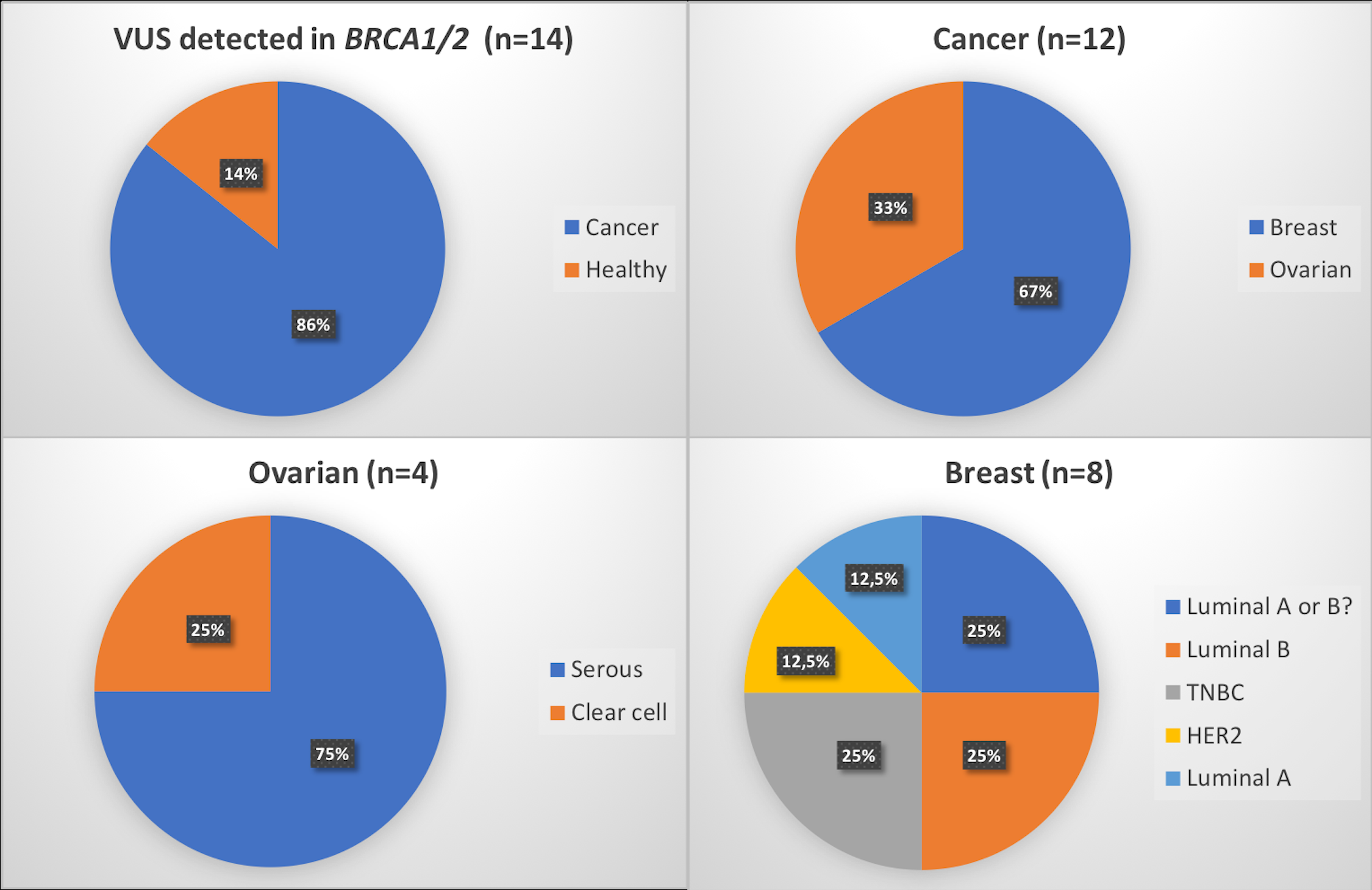

Supplement: S5 Fig — (TIF) [file pone.0201086.s006.tif]

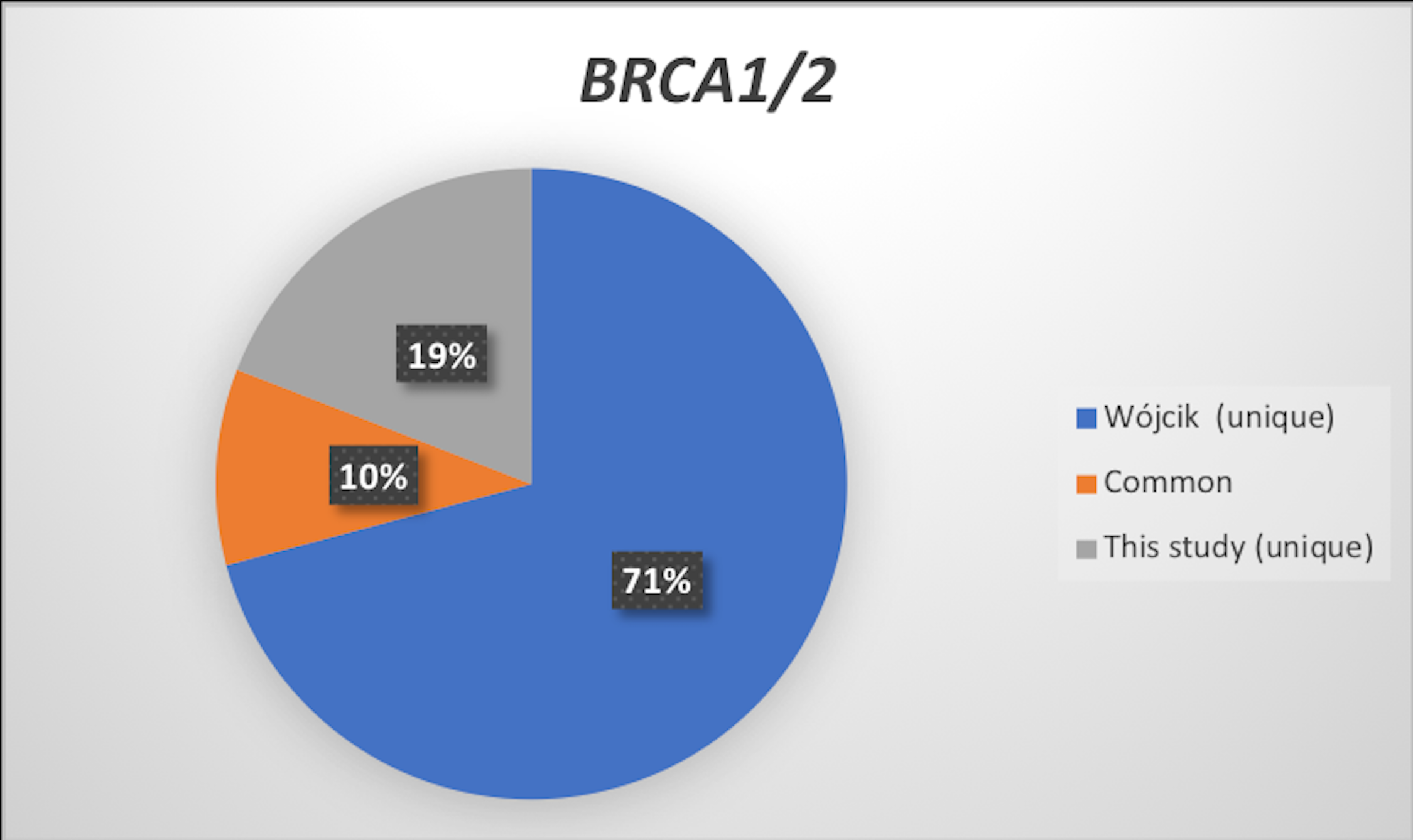

Supplement: S6 Fig — (TIF) [file pone.0201086.s007.tif]
